# Respiratory syncytial virus infection: an innate perspective

**DOI:** 10.12688/f1000research.9637.1

**Published:** 2016-12-21

**Authors:** Cecilia Johansson

**Affiliations:** 1 Respiratory Infections Section, St Mary's campus, National Heart and Lung Institute, Imperial College London, London, W2 1PG, UK

**Keywords:** RSV, respiratory syncytial virus, lower respiratory disease, immune response to RSV, immune-mediated virus clearance, immunopathology

## Abstract

Respiratory syncytial virus (RSV) is a common cause of upper respiratory tract infection in children and adults. However, infection with this virus sometimes leads to severe lower respiratory disease and is the major cause of infant hospitalisations in the developed world. Several risk factors such as baby prematurity and congenital heart disease are known to predispose towards severe disease but previously healthy, full-term infants can also develop bronchiolitis and viral pneumonia during RSV infection. The causes of severe disease are not fully understood but may include dysregulation of the immune response to the virus, resulting in excessive recruitment and activation of innate and adaptive immune cells that can cause damage. This review highlights recent discoveries on the balancing act of immune-mediated virus clearance versus immunopathology during RSV infection.

## Introduction

**Figure 1.**
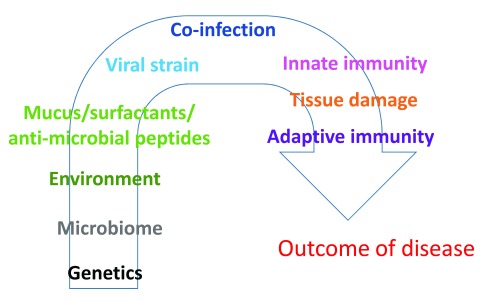
Possible determinants of severity of disease during respiratory syncytial virus (RSV) infection. Many host, environmental, or viral factors can determine the outcome and severity of RSV disease. Most likely an interplay of several factors will determine why some patients develop severe disease.

Respiratory syncytial virus (RSV) is the most common trigger of bronchiolitis and viral pneumonia, especially in infants, and there are links between severe RSV disease and later development of asthma and wheeze ^1– 3^. There are at present no effective RSV anti-virals or RSV vaccines in the clinic; therefore, infection with the virus remains a clinical problem worldwide, and avoiding the development of severe lower respiratory tract infection constitutes an unmet need. There are many known risk factors for severe RSV disease such as pre-term birth, lung underdevelopment, and congenital heart disease ^4, 5^. However, previously healthy babies lacking any of the above risk factors are also admitted to hospital with severe lower respiratory tract RSV infection ^1– 3, 6^. Possible parameters determining the severity of disease include genetic susceptibility of the host, presence of co-infections with other pathogens, viral genotype, and viral load ( Figure 1 ^4– 6^). However, other reasons relate to how immune responses to the virus are induced and regulated, an area about which we still know very little ( Figure 1). Innate immune responses occur immediately upon infection and are important for the early containment of pathogens before adaptive immune responses (antibodies and T cells) can be mobilised. They also direct subsequent adaptive immune responses and dictate how strongly the host responds to the invading pathogen. Innate immune responses are difficult to investigate during natural RSV infection, especially in children, as they have generally waned by the time of hospital visit/admission. However, experimental models of RSV infection can be used to begin to understand how innate immunity to the virus is elicited and impacts disease progression. In this commentary, recent advances in understanding RSV infection are summarised, with a focus on new findings in the area of innate immunity to the virus.

## RSV infection

RSV is a negative sense, single-stranded RNA virus of the Pneumoviridae family (previously classified in the Paramyxoviridae family ^7, 8^). It was first described in chimpanzees in 1955 ^9^ and shortly thereafter detected in children with respiratory illness ^10^. RSV is estimated to cause 34 million episodes of lower respiratory tract infections leading to 3.4 million hospitalisations and up to 199,000 deaths per year in children younger than 5 years of age ^11^. Hospitalisation is most common in infants between 2 and 6 months of age ^6^.

RSV infects the respiratory tract by initially binding to molecules on the apical surface of epithelial cells or by non-specific uptake via macropinocytosis ^7, 12^. Which receptors are involved in binding the virus and facilitating infection is not fully elucidated, but several cell surface molecules have been implicated in the process. For example, glycosaminoglycans expressed on cell surfaces can bind to the envelope glycoproteins of RSV, namely the G and F proteins. RSV G is important for viral attachment to the host cells, while RSV F is involved in the fusion of the viral envelope with either the cell plasma membrane or the delimiting membrane of macropinosomes ^7, 12^. RSV F protein expressed on the surface of neighbouring infected cells also causes their fusion to form syncytia, a characteristic feature of the infection that lends the virus its name ^7^. RSV F protein also binds the cellular protein nucleolin and this increases infection ^13^. In addition, CX3CR1 (the fractalkine receptor) was recently shown to be expressed on ciliated epithelial cells and can bind to RSV G ^14– 16^, since RSV G contains a CX3C motif ^17, 18^. Notably, mice lacking CX3CR1 are less susceptible to RSV infection ^15^, underscoring the importance of this interaction in viral entry. Following attachment, and fusion, RSV enters the cytoplasm and the replication cycle ensues. Progeny viruses eventually assemble and bud off the plasma membrane after the formation of long protruding structures called filaments ^7^. Released viral particles then infect neighbouring cells and propagate the infectious process.

Whether lower airway disease is caused by uncontrolled virus infection resulting in syncytial cell death and epithelial barrier breakdown or whether it is due to tissue damage caused by a dysregulated immune response (immunopathology) is not fully understood. Importantly, the two are not independent variables. A high viral load has been associated with high release of pro-inflammatory immune mediators and more severe symptoms ^19– 23^. Thus, it is possible that the development of severe disease is due to an early lack of control of the virus, which leads to epithelial cell damage and a high release of pro-inflammatory mediators that recruit and activate leukocytes in the lung and induce an excessive immune response that results in immunopathology ^20, 24– 26^. The risk groups for severe RSV disease are the young (less than one year of age) and the old (more than 65 years of age) ^27, 28^. Infants have an immature immune system, which renders them less able to mount an efficient anti-viral response ^28, 29^. In addition, it is likely that structural features including small airway calibre may make infants more prone to critical airway narrowing and resultant hypoxia in the face of lung inflammation ^28^. The elderly have a senescing immune system and are therefore less able to induce appropriate responses to invading pathogens ^30, 31^. It is possible that the innate immune response in these two at-risk groups is suboptimal, which results in an unbalanced immune response and inefficient balance between viral control and immunopathology.

## Innate immune responses to RSV

RSV research has long been focused on adaptive immunity. Recently, the importance of innate immunity has been highlighted, especially from studies using animal models. There are many factors that can influence the development and severity of disease ( Figure 1). The first lines of defence are mucus ^32^, anti-microbial peptides ^33^, and surfactants ^34, 35^. The local lung microbiota can also most likely influence RSV infection rate and the immune response to the virus, but this is an emerging concept for which, at present, there are limited supporting data. However, it has recently been suggested that the nasopharyngeal microbiota in young children can influence the spread of the infection to the lower airways and modulate the host immune response to RSV infection ^36, 37^. It remains to be elucidated if the composition of the microbiota is changed by the infection or if a specific composition of microbiota is determining the degree and spread of infection.

The next layer of defence the virus has to confront is the resident cells of the respiratory tract, mainly epithelial cells, alveolar macrophages (AMs), dendritic cells (DCs), and innate lymphoid cells. Many cells express pattern recognition receptors (PRRs) that can bind to pathogen-associated molecular patterns (PAMPs) and signal to initiate the production of pro-inflammatory cytokines and chemokines that serve to orchestrate anti-viral immunity. Some of these have potent anti-viral effects themselves, such as the type I interferons (IFN-α/β) ^38, 39^. These cytokines are transiently produced and bind to the type I IFN receptor (IFNAR) expressed on all nucleated cells to signal to induce the expression of a large number of proteins that help restrict viral replication. More recently, it has become apparent that type I IFNs also play a key role in inducing cytokines and chemokines that promote the recruitment and activation of immune cells ^24, 25, 38^. As a counter-strategy, many viruses have evolved proteins that hinder type I IFN production or block IFNAR signalling. RSV has two non-structural proteins, NS1 and NS2, that inhibit type I IFN production and signalling in infected cells ^40^ as well as interfere with epithelial cell sloughing ^41^. In addition, RSV N protein has been suggested to also be able to inhibit IFN-β induction ^42^. Furthermore, viruses, including RSV, can subvert the cell-intrinsic anti-viral responses by manipulating microRNA generation and/or function ^43, 44^.

Genetic analyses of infants show an association of single nucleotide polymorphisms (SNPs) in genes encoding type I IFNs or proteins involved in IFNAR signalling with severe RSV disease ^45, 46^. Also, a deficiency in type I IFN production by cells from infants ^47^ and from neonatal mice ^48, 49^ has been shown. In contrast, some studies show a higher level of IFN-α in nasopharyngeal wash in more severely sick infants compared to controls ^50^. It is possible that the type I IFN response is not detectable by the time children are admitted to hospital with lower respiratory tract RSV infection, since this is likely to happen several days after the initial infection, at which time the production of type I IFNs is declining and these cytokines are difficult to detect.

Cytosolic PRRs of the RIG-I-like receptor (RLR) family that signal via mitochondrial anti-viral signalling protein (MAVS) are crucial for the production of type I IFNs and other pro-inflammatory cytokines during RSV infection ^24, 26, 51^. UV-inactivated RSV does not elicit type I IFN responses ^24, 52^, but defective RSV genomes can in both mice and humans ^53^. Interestingly, the major sources of type I IFNs in the lower airways of mice during experimental RSV infection are AMs ^24^, and they use cytosolic MAVS-coupled sensors to induce this production ^24, 52^. However, these cells are not productively infected by RSV ^52^.

Several Toll-like receptors (TLRs), such as TLR2, 3, 4, and 7, are also implicated in the recognition of RSV ^54, 55^. For example, RSV F can bind TLR4 ^56^ and SNPs in the TLR4 gene correlate with severe RSV disease ^57– 59^. However, mouse models show variable dependency on TLR4 for the development of disease ^56, 60, 61^. TLR4 is best known as a receptor for lipopolysaccharide (LPS), a bacterial product, and a recent study showed that an intersection of TLR4 genotype with LPS content in the home environment determines the severity of RSV disease ^62^. This indicates that the interplay between genetics and the environment, including the microbiota and co-infections, will be part of the severity of disease caused by RSV ( Figure 1). Interestingly, even mice genetically lacking the ability to signal via all TLRs and RLRs are able to control RSV infection and mount T cell responses to the virus ^63^. This suggests that additional mechanisms for detecting RSV infection exist that can compensate for the lack of PRR signalling. Like PAMPs, damage-associated molecular patterns (DAMPs) released by dead cells can trigger immunity ^64^. One part of RSV disease manifestation is small airway obstruction caused by a mix of mucus, infiltrating cells, and dead or dying epithelial and inflammatory cells ^1, 6^. Many DAMPs will therefore at this stage be expected to be present freely in the lungs and might contribute to the initiation of immune responses to the virus. Recently, RSV infection was shown to trigger the release of DAMPs such as high mobility group box 1 (HMGB1) ^65^ and S100A9 ^66^.

PAMP or DAMP recognition often results in the production of pro-inflammatory cytokines and chemokines, many of which, including TNF, IL-6, and CCL2, have been associated with severe lower respiratory tract infection and recurrent wheeze ^6, 67^. For example, recently the epithelial-derived cytokine IL-33 was shown to contribute to disease severity in neonates and was also found in nasal aspirates from infants after RSV infection ^68^. Locally produced cytokines are important for lung cell proliferation, activation, and differentiation, and chemokines are important for orchestrating immune cell infiltration into the lungs. One of the first cell types to be recruited after lung infections is neutrophils. They infiltrate the lung in vast numbers during RSV infection ^25, 69– 71^ and have multiple functions such as phagocytosis, production of reactive oxygen species, and secretion of proteolytic enzymes ^72^. Activated neutrophils can also form neutrophil extracellular traps (NETs), networks of DNA and microbicidal proteins, that can capture RSV ^73, 74^. However, whether neutrophils are beneficial for viral control or, rather, become a cause of lung injury/occlusion during RSV infection needs further investigation ^72^. The next cell type to arrive in the lung is the monocytes. Type I IFNs are instrumental for recruiting inflammatory monocytes by inducing the production of monocyte chemoattractants such as CCL2. The monocytes are important for controlling the virus and have recently been shown to contribute to viral clearance/control of RSV in the lower airways of mice ^24^. Interestingly, children with bronchiolitis display high levels of CCL2 in nasopharyngeal wash ^50^ and in bronchoalveolar lavage (BAL) fluid ^75, 76^. In the mouse model of RSV infection, lung monocytes are anti-viral and contribute to host protection ^24^, while in a model of influenza virus infection monocytes cause pathology ^77, 78^. It is interesting to speculate that increased/uncontrolled levels of type I IFNs could recruit high numbers of monocytes that are initially important for viral control but, in excess, become detrimental and contribute to lung immunopathology.

Natural killer (NK) cells and, subsequently, T cells infiltrate the lungs following neutrophils and monocytes. Both of these cell types have an anti-viral effect during RSV infection ^79– 81^. Each infiltrating cell type has a role in anti-viral defence, and its recruitment is well orchestrated in order to clear the virus while limiting tissue damage, a particularly important issue in delicate tissues, such as the lung, which needs to preserve gas exchange function irrespective of ongoing infectious challenge. If lung recruitment of immune cell types is dysregulated, the balance of viral control versus tissue damage is lost, and pathology and severe disease can ensue. Thus, innate immune responses to RSV are crucial in executing the initial control of the virus and in directing a balanced immune response.

## Adaptive immune responses to RSV

DCs are key in the crosstalk between innate and adaptive immunity ^82^. They are activated by PRR signalling and pro-inflammatory mediators in the lung to increase their antigen processing and presentation capability and migrate to regional lymph nodes where they convey viral antigens to naïve T and B cells ^83^. It is interesting to note that there are fewer DCs in the lungs and lymph nodes of infants and they are also less functional after RSV infection compared to those from adult lungs ^49, 84, 85^. Also, fewer and less functional plasmacytoid DCs are found in young children ^47, 86^. Flt3 ligand subcutaneous administration to neonatal mice mobilises more lung DCs and results in less lung inflammation after re-infection with RSV ^49^. Altogether, these data suggest that a correct DC response is vital for a balanced immune response during RSV infection.

RSV-specific B and T cells are activated in the lymph nodes and proliferate, migrate, and start executing their respective functions a few days after the start of the RSV infection. The antibodies secreted by RSV-specific B cells are important to prevent viral spread and reinfection. Interestingly, RSV-specific antibodies have very short half-life and serum titres, and the number of IgA ^+^ memory B cells decreases with time ^87– 89^. This is unlike other respiratory infections, such as with influenza virus, in which antibodies persist and confer lifelong protection against the original infecting strain. However, nasal anti-RSV IgA levels correlate with protection from experimental infection in adults ^89^, while levels of nasal (maternally derived) anti-RSV IgG correlate with lower viral load in infants ^90^. Furthermore, anti-RSV antibodies have been found in amniotic fluid ^91^ and could potentially be protective to the lungs of newborns. Altogether, these data indicate a possible beneficial role for antibodies during RSV infection but raise the conundrum of why protective antibody-dependent responses fail to establish memory in adults, permitting re-infection with the same RSV strain.

T cells arrive in the lungs from the lymph nodes a few days after the start of RSV infection. They are important for viral clearance during primary infection ^92^, and protective responses to RSV infection are characterised by a T helper 1 (Th1)-dominated response with T cells that produce IFN-γ (CD4 ^+^ and CD8 ^+^ T cells) and kill infected cells with perforin and granzyme B (CD8 ^+^ T cells) ^93^. In contrast, if the T cell responses are skewed towards a Th2 type, they can contribute to immunopathology ^62, 93– 95^. Although, more recently, some of these observations have also been attributed to Th17 induction ^96^.

After a first infection with RSV, memory T cells are generated and can be mobilised when the host re-encounters RSV at a later stage. There are different types of memory T cells: central memory, effector memory, and tissue-resident memory (T _RM_) T cells. RSV-specific T _RM_ cells localise to the lungs and can exert an innate-like function when reinfection occurs ^97, 98^. T _RM_ cells have been found in mouse ^99^ and human ^100^ lungs after RSV infection has cleared. Even though memory T cell responses develop during RSV infection, their longevity has been debated, as re-infections with the same virus strain occur throughout life. Also, the type of memory T cells (Th1, Th2, or Th17) is important for the outcome of the reinfection, with an increased Th2 or Th17 response correlating with enhanced disease ^93^. Increasing evidence suggests that T _RM_ cells are crucial for protection from re-infection with various pathogens and therefore an important consideration for vaccine development (see below). Future research will inform a more precise role of T _RM_ cells during RSV infection.

A strong or dysregulated T cell response to RSV will be detrimental to lung tissue integrity. CD8 ^+^ and CD4 ^+^ T cells start to upregulate IL-10 production during RSV infection ^101– 104^ most likely in order to dampen the ongoing immune response, as IL-10 can have anti-inflammatory effects ^105^. Also, T regulatory cells (Tregs) are important for keeping the T-cell-driven inflammation in check, especially the Th17 and Th2 types, during RSV infection ^96, 106– 112^. Thus, adaptive immune responses to RSV are important for the final viral clearance and for a rapid memory response in case of a re-infection, but this response also has to be under tight control in order to limit immunopathology.

## Potential vaccines

To date, the treatment of RSV disease is mainly supportive and no specific anti-virals or vaccines are currently licenced ^1, 113^. It has proven difficult to achieve viral control without causing immunopathology. In the 1960s, a trial using formalin-inactivated RSV failed to induce protection in vaccinated children but instead enhanced disease after natural infection with RSV ^114^. An issue for vaccine development is the lack of correlates of protection coupled to the difficulties of lung sampling, which is crucial as protective immune responses to RSV will not necessarily be evident in the blood ^99, 100^. Despite these issues, several vaccines (subunit, live-attenuated, and vector vaccines) and routes of vaccination are currently being developed and tested ^113, 115, 116^. In addition, the adjuvants used for triggering the innate immune responses during vaccination are an important avenue of ongoing research to find an effective vaccine. Passive immunisation has also proven useful, and Palivizumab, a monoclonal antibody against RSV F protein, is given to high-risk infants to prevent infection and the development of severe disease ^117^. Recently, an anti-RSV G monoclonal antibody was shown to be more effective than anti-F in preventing RSV disease in animal models ^118^, but this has yet to be tested in humans. Finally, maternal vaccination against RSV is an interesting future avenue for generating protection in the first month of life in newborns via placental or breast milk transfer of maternal RSV-specific antibodies to the infant ^119^.

## Conclusions

The understanding of the causes and mechanisms of RSV disease has increased tremendously over the last few years. However, many unknown factors are yet to be discovered. The influence of the microbiota and environment on the severity of disease as well as the understanding of the specific roles of individual lung and immune cells during the infection are still waiting to be unveiled. The knowledge of basic mechanisms will be instrumental for the understanding of disease progression, outcome, and severity and will more efficiently guide vaccine and therapy development in the future.

## Abbreviations

AMs, alveolar macrophages; BAL, bronchoalveolar lavage; DAMPs, damage-associated molecular patterns; DCs, dendritic cells; IFNAR, type I interferon receptor; IFNs, interferons; LPS, lipopolysaccharide; MAVS, mitochondrial anti-viral signalling protein; PAMPs, pathogen-associated molecular patterns; PRRs, pattern recognition receptors; RLRs, RIG-I-like receptors; RSV, respiratory syncytial virus; SNP, single nucleotide polymorphism; Th, T helper; TLRs, Toll-like receptors; T _RM_ cells, tissue-resident memory T cells.
